# Identification of predictors and construction of a prediction model for the quality of life in laryngeal carcinoma patients in China using revised core nursing outcomes

**DOI:** 10.1186/s12912-024-02539-y

**Published:** 2024-12-02

**Authors:** Yongxia Ding, Yanzhi Tian, Ruirui Duan, Jing Xu, Huixian Yang, Jinxia Xu, Liyun Tang

**Affiliations:** 1 Nursing College of Shanxi Medical University, Taiyuan, Shanxi China; 2 Shanxi Key Laboratory of Otolaryngology, Head and Neck Cancer, Taiyuan, Shanxi China; 3 Nursing Management and Health Promotion Research Center, Shanxi Medical University, Taiyuan, Shanxi China; 4 Department of Suegery, Shanxi Cancer Hospital, Taiyuan, Shanxi China; 5 Department of Nursing, the First Affiliated Hospital, Jinzhou Medical University, Jinzhou, Liaoning China

**Keywords:** Core postoperative nursing outcomes, Quality of life, Laryngeal carcinoma, Prediction model

## Abstract

**Background:**

This study aimed to identify potential predictors and construct a predictive model for the quality of life (QoL) in patients with laryngeal carcinoma (LC) using the revised core nursing outcomes.

**Methods:**

We recruited 331 LC patients underwent laryngectomy between March 2018 and March 2022 from three hospitals. The revised core nursing outcomes contained 14 items in the “Physiologic Health,” “Psychosocial Health,” “Health Knowledge & Behavior,” and “Perceived Health”, while the QoL was assessed using the Chinese version of the Functional Assessment of Cancer Therapy-Head and Neck. Potential predictors of QoL were explored using logistic regression analysis, and odds ratio (OR) with 95% confidence interval (CI) was applied as effect estimates. A prediction model was constructed to predict QoL using the receiver operating characteristic (ROC) curve, and the predictive value was assessed using the area under the ROC curve (AUC).

**Results:**

Of the 331 included patients, 137 had a poor QoL. After adjusting for potential confounding factors, we noted female sex (OR: 10.91; 95%CI: 1.24–96.14; *P* = 0.031), and alcohol consumption (OR: 4.55; 95%CI: 1.32–14.29; *P* = 0.017) were associated with an elevated incidence of poor QoL, while age 50.0–65.0 years (OR: 0.02; 95%CI: 0.00-0.15; *P* < 0.001), junior high school as the highest level of schooling (OR: 0.10; 95%CI: 0.03–0.33; *P* < 0.001), living in cities and towns (OR: 0.07; 95%CI: 0.02–0.37; *P* = 0.002), and partial laryngectomy (OR: 0.10; 95%CI: 0.03–0.41; *P* = 0.001) were associated with a lower incidence of poor QoL. Moreover, Physiologic Health score (OR: 1.16; 95%CI: 1.06–1.27; *P* = 0.001), Psychosocial Health score (OR: 0.48; 95%CI: 0.39–0.58; *P* < 0.001), Health Knowledge & Behavior score (OR: 0.92; 95%CI: 0.86–0.97; *P* = 0.006), and Perceived Health score (OR: 0.36; 95%CI: 0.25–0.52; *P* < 0.001) were associated with the incidence of QoL in LC patients. The constructed prediction model based on these factors had an AUC for predicting QoL of 0.96 (95% CI: 0.94–0.98).

**Conclusions:**

This study found age, sex, educational status, residential location, alcohol consumption, surgical approach, and the revised core nursing outcomes are significantly associated with QoL in LC patients. The predictive value of the constructed model was high, which suggesting the clinical nurses should pay attention to the evaluation of postoperative care outcomes in order to enhance QoL.

## Background

Laryngeal carcinoma (LC) accounts for nearly 30-40% of head and neck malignancies [1]. Although there has been a decline in the global incidence of LC, it is the second most common head and neck malignancy, and remains a serious health issue [2–4]. Thyroid squamous cell carcinoma has a much higher incidence rate in the northern and northeastern regions of China than in the provinces south of the Yangtze River [5]. Early-stage LC is treated with chemoradiotherapy, and the most widely used treatment option for advanced LC is surgery, including total and partial laryngectomy [6]. However, laryngectomy is associated with poor quality of life (QoL) due to the use of tubes and the associated loss of speech [7, 8].

Nursing outcomes, or patient outcomes influenced by nursing care, refer to the observable behaviors or subjective perceptions of individuals, families, or communities that can be assessed along a spectrum in response to nursing interventions. The Nursing Outcomes Classification (NOC), developed by the University of Iowa, is widely used to assess patient and client outcomes during nursing care. The core nursing outcome is a set of concise outcomes that reflects the essence of specialized nursing practice and effectively guides specialized nursing practice. In our previous study, we evaluated and revised the suitable postoperative core nursing outcomes for LC patients in China [9]. However, the association between postoperative core nursing outcomes and the QoL of patients after laryngectomy remained unclear. Therefore, this study applied the revised postoperative core nursing outcomes from the “Core Nursing Outcomes for Otorhinolaryngology Head-Neck” for LC patients in China and assessed the association between the revised core nursing outcomes and QoL of LC patients. Furthermore, a prediction model for QoL of patients with LC was constructed using the revised core nursing outcomes.

## Methods

### Patients

This study was approved by the Ethics Committee of the College of Nursing, Shanxi Medical University (2020sll002). The study protocol was conducted according to the principles expressed in the Declaration of Helsinki. This study was reported in accordance with the STROBE Reporting Statement. A total of 331 patients who underwent laryngectomy for LC were recruited between March 2018 and March 2022 from three hospitals in northern and northeastern regions of China. The inclusion criteria were as follows: (1) pathologically confirmed LC treated by total or partial laryngectomy, and (2) ability to speak and understand the questionnaire content correctly. Patients with other tumors or mental illnesses, those who received chemoradiotherapy before undergoing laryngectomy, and those who had recurrent or metastatic LC within 6 months were excluded.

### Data collection

The variables of interest included sex, age, educational status, residential location, occupation, marital status, income, smoking status, alcohol consumption, surgical approach, and tube use. The general information questionnaire was completed by members of the research group as investigators, and the patients were instructed to complete the questionnaire 1 day before discharge. Questionnaires were immediately collected. If it was inconvenient for the patients to fill in the questionnaire themselves, the investigator dictated the contents of the entry, the patient answered, and the investigator wrote on behalf of the patient.

### Revised core nursing outcomes

The revised postoperative core nursing outcomes in patients with LC in China was described in our previous study [9]. In summary, 130 nurses in the otorhinolaryngology departments of 10 hospitals were employed for the initial inclusion of the core nursing outcomes based on their degree of use [10]. The expert group method was then used to screen indicators in clinical practice. The improved Delphi method for expert consultation was used to revise the core nursing outcomes and related indicators [11]. The revision process applied expert group and Delphi expert consultation methods [9]. Subsequently, we identified 14 postoperative core nursing outcomes in domains of “Physiologic Health,” “Psychosocial Health,” “Health Knowledge & Behavior”, and “Perceived Health.” The Kronbach α coefficient was applied to assess reliability, and the Kronbach α coefficients for “Physiologic Health,” “Psychosocial Health,” “Health Knowledge & Behavior,” and “Perceived Health” were 0.989, 0.765, 0.994, and 0.806, respectively. The validities of the revised core nursing outcomes for “Physiologic Health,” “Psychosocial Health,” “Health Knowledge & Behavior”, and “Perceived Health” were 0.968, 0.796, 0.882, and 0.861, respectively.

### QoL assessment

The postoperative QoL of LC patients was assessed using the Chinese version of the Functional Assessment of Cancer Therapy-Head and Neck (FACT-H&N) [12]. The FACT-H&N scale consists of specific modules of head and neck malignancies and common modules in cancer for assessing QoL and includes five dimensions (physical activity, social conditions, emotional state, functional status, and additional attention) including 38 items. Each item was set in a hierarchical style, and the score for each item ranged from 0 to 4, with a total score of 0 to 152. Higher scores indicated better QoL. If a score was not available for a specific item, the score for that dimension was calculated by adding the scores of each item in the dimension and multiplying by the number of items in the dimension divided by the actual number of items answered.

### Statistical analysis

Patient characteristics according to QoL are presented as mean (standard deviation) or median (quartile) for continuous variables based on data distribution, and frequencies and proportions are used to describe categorical variables. The independent t-test, Kruskal-Wallis test, and Chi-square test were used to assess the differences between groups. Potential predictors of QoL were explored using a univariate logistic regression analysis, and predictors of QoL with *P* < 0.05 were selected as potential covariates for the multivariate model. Subsequently, a multivariate logistic regression model was constructed to identify potential predictors of QoL, and the final set of predictors was identified using stepwise selection with a threshold of *P* < 0.05. Odds ratios (ORs) with 95% confidence intervals (CIs) were used to estimate effects. Physiological Health, Psychosocial Health, Health Knowledge & Behavior, and Perceived Health in the revised core nursing outcomes are mandatorily incorporated into the multifactorial model. The predictive value of the constructed model was assessed using the area under the receiver operating characteristic curve (AUC). All tests were two-sided, and *P* < 0.05 was regarded as statistically significant. IBM SPSS Statistics for Windows, version 19.0 (SPSS 19.0) was used to perform the statistical analysis in our study.

## Results

### Patient characteristics

A total of 331 patients were enrolled, of whom 310 (93.66%) were men. A total of 315 (95.17%) patients had a smoking history, and 53 (16.01%) had a history of alcohol consumption. A total of 119 (35.95%), 193 (58.31%), and 19 (5.74%) patients underwent total laryngectomy, partial laryngectomy, and laryngoscopy, respectively. The patient characteristics are summarized in Table 1. The QoL scores included physical activity (14.00), social conditions (16.00), emotional state (10.00), functional status (11.00), and attention (27.00). There were significant differences between the good and poor QoL groups for sex (*P* = 0.004), occupation (*P* = 0.009), and marital status (*P* = 0.003).

**Table 1:** The baseline characteristics of included patients

Variables	Overall (*n* = 331)	QoL		*P* value
		Good (*n* = 194)	Poor (*n* = 137)	
**Age (years)**				0.174
< 50.0	23 (6.95)	16 (8.25)	7 (5.11)	
50.0–65.0	188 (56.80)	115 (59.28)	73 (53.28)	
> 65.0	120 (36.25)	63 (32.47)	57 (41.61)	
**Sex**				0.004
Male	310 (93.66)	188 (96.91)	122 (89.05)	
Female	21 (6.34)	6 (3.09)	15 (10.95)	
**Educational status**				0.105
Primary schools	104 (31.42)	55 (28.35)	49 (35.77)	
Junior high school	145 (43.81)	86 (44.33)	59 (43.07)	
High school or above	82 (24.77)	53 (27.32)	29 (21.17)	
**Residential location**				0.252
Village	219 (66.16)	123 (63.40)	96 (70.07)	
Cities and towns	112 (33.84)	71 (36.60)	41 (29.93)	
**Occupation**				0.009
Farmer	233 (70.39)	129 (66.49)	104 (75.91)	
Worker	77 (23.26)	56 (28.87)	21 (15.33)	
Other	21 (6.34)	9 (4.64)	12 (8.76)	
**Marital status**				0.003
Married	318 (96.07)	181 (93.30)	137 (100.00)	
Unmarried	9 (2.72)	9 (4.64)	0 (0.00)	
Verwitwet	4 (1.21)	4 (2.06)	0 (0.00)	
**Smoking**				0.648
No	16 (4.83)	8 (4.12)	8 (5.84)	
Yes	315 (95.17)	186 (95.88)	129 (94.16)	
**Alcohol**				0.894
Yes	53 (16.01)	32 (16.49)	21 (15.33)	
No	278 (83.99)	162 (83.51)	116 (84.67)	
**Surgery approach**				0.114
Total laryngectomy	119 (35.95)	61 (31.44)	58 (42.34)	
Partial laryngectomy	193 (58.31)	122 (62.89)	71 (51.82)	
The laryngoscope was removed	19 (5.74)	11 (5.67)	8 (5.84)	
**The escrow**				1.000
No	19 (5.74)	11 (5.67)	8 (5.84)	
Yes	312 (94.26)	183 (94.33)	129 (94.16)	
Physiologic Health	171.00 (166.00,178.00)	171.00 (164.00,177.00)	175.00 (167.00,182.00)	0.054
Vital Signs	25.00 (25.00,25.00)	25.00 (25.00,25.00)	25.00 (24.00,25.00)	0.012
Respiratory Status	37.00 (36.00,39.00)	36.00 (35.00,39.00)	38.00 (37.00,40.00)	< 0.001
Infection Severity	26.00 (24.00,29.00)	26.00 (24.00,28.00)	27.00 (24.00,29.00)	0.138
Communication	12.00 (7.00,15.50)	11.00 (7.00,14.75)	14.00 (6.00,17.00)	0.074
Swallowing Condition: Pharyngeal Phase	35.00 (32.00,37.00)	35.00 (32.00,38.00)	35.00 (34.00,37.00)	0.848
Nutritional Status	12.00 (11.00,12.00)	12.00 (11.00,13.00)	12.00 (12.00,12.00)	0.074
Tissue Integrity	27.00 (26.00,27.00)	27.00 (26.00,27.00)	27.00 (25.00,27.00)	0.684
Psychosocial Health	30.00 (25.00,35.00)	34.00 (30.00,36.00)	25.00 (22.00,27.00)	< 0.001
Health Status	13.00 (10.50,16.00)	15.00 (13.00,16.00)	11.00 (9.00,13.00)	< 0.001
Will to Live	13.00 (12.00,16.00)	16.00 (13.00,18.00)	12.00 (9.00,13.00)	< 0.001
Adaptation to Physical Disabilities	3.00 (3.00,3.00)	3.00 (3.00,3.00)	3.00 (2.00,3.00)	0.002
Health Knowledge & Behavior	41.00 (35.00,47.00)	46.00 (41.00,50.00)	36.00 (33.00,39.00)	< 0.001
Infection Management	7.00 (6.00,9.00)	7.00 (6.00,9.00)	7.00 (5.00,8.00)	0.011
Smoking Cessation Behavior	11.00 (9.00,14.00)	13.00 (10.00,16.00)	9.00 (8.00,11.00)	< 0.001
Aspiration Preventing	22.00 (19.00,27.00)	26.00 (20.25,28.00)	20.00 (19.00,22.00)	< 0.001
Perceived Health	17.00 (15.00,18.00)	17.00 (15.00,18.00)	17.00 (13.00,18.00)	0.075
Physical activity	14.00 (13.00,16.00)	14.00 (13.00,16.00)	14.00 (13.00,17.00)	0.083
Social conditions	16.00 (13.00,20.00)	18.50 (16.00,22.00)	13.00 (12.00,15.00)	< 0.001
Emotional state	10.00 (9.00,13.00)	9.50 (8.00,12.00)	11.00 (10.00,13.00)	< 0.001
Functional status	11.00 (9.00,17.00)	17.00 (12.00,18.00)	9.00 (7.00,10.00)	< 0.001
Additional attention	27.00 (22.00,30.00)	30.00 (27.00,31.00)	22.00 (18.00,24.00)	< 0.001

We also examined the scores of the revised postoperative core nursing outcomes of the patients with LC. The score for Physiologic Health was 171.00, and it consisted of the scores for 0802 Vital Signs (25.00), 0415 Respiratory Status (37.00), 0703 Infection Severity (26.00), 0902 Communication (12.00), 1013 Swallowing Condition: Pharyngeal Phase (35.00), 1004 Nutritional Status (12.00), and 1101 Tissue Integrity: Skin & Mucous Membrane (27.00). The score for Psychosocial Health was 30.00, and it consisted of the scores for 1300 Acceptance: Health Status (13.00), 1206 Will to Live (13.00), and 1308 Adaptation to Physical Disabilities (3.00). The score for Health Knowledge & Behavior was 41.00, and it consisted of the scores for 1842 Knowledge: Infection Management (7.00), 1625 Smoking Cessation Behavior (11.00), and 1918 Aspiration Prevention (22.00). The score for Perceived Health was 17.00, and it consisted of the score for 2102 Pain Level. We noted no significant differences between the good and poor QoL groups for Physiologic Health (*P* = 0.054) and Perceived Health (*P* = 0.075), whereas potential significant differences between groups for Psychosocial Health (*P* < 0.001) and Health Knowledge & Behavior (*P* < 0.001).

### Factors influencing QoL

**Fig. 1:**
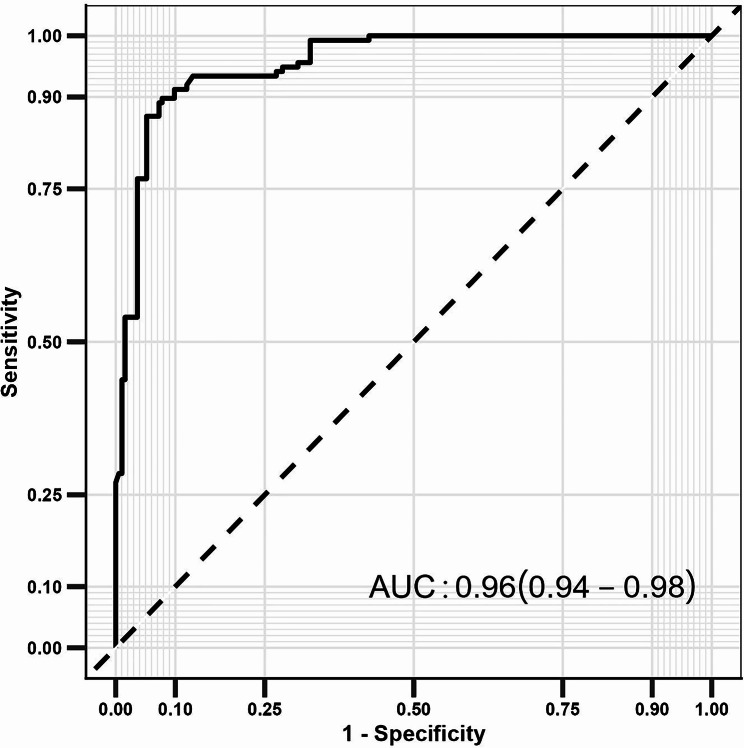
Receiver operating curve of the ten-component risk factor model for predicting QoL (area under the curve = 0.96). QoL: quality of life

The predictors of QoL are shown in Table 2. The univariate logistic regression analysis found that female sex (OR: 4.62; 95% CI: 1.64–13.05; *P* = 0.004) was associated with an elevated incidence of poor QoL, while worker (OR: 0.47; 95% CI: 0.27–0.82; *P* = 0.008), unmarried or other marital status (OR: 0.07; 95% CI: 0.00-0.35; *P* < 0.001), and partial laryngectomy (OR: 0.61; 95% CI: 0.39–0.97; *P* = 0.038) were associated with lower incidence of poor QoL. Moreover, increased Psychosocial Health score (OR: 0.72; 95% CI: 0.67–0.77; *P* < 0.001), Health Knowledge & Behavior score (OR: 0.87; 95% CI: 0.83–0.90; *P* < 0.001), and Perceived Health score (OR: 0.86; 95% CI: 0.78–0.95; *P* = 0.003) were associated with lower incidence of poor QoL, while Physiologic Health score did not affect QoL. After adjusting for potential confounding factors, age 50.0–65.0 years (OR: 0.02; 95%CI: 0.00-0.15; *P* < 0.001), female sex (OR: 10.91; 95%CI: 1.24–96.14; *P* = 0.031), junior high school as the highest level of schooling (OR: 0.10; 95%CI: 0.03–0.33; *P* < 0.001), living in cities and towns (OR: 0.07; 95%CI: 0.02–0.37; *P* = 0.002), alcohol consumption (OR: 4.55; 95%CI: 1.32–14.29; *P* = 0.017), and partial laryngectomy (OR: 0.10; 95%CI: 0.03–0.41; *P* = 0.001) were associated with the incidence of poor QoL. In addition, increased Psychosocial Health score (OR: 0.48; 95%CI: 0.39–0.58; *P* < 0.001), Health Knowledge & Behavior score (OR: 0.92; 95%CI: 0.86–0.97; *P* = 0.006), and Perceived Health score (OR: 0.36; 95%CI: 0.25–0.52; *P* < 0.001) were associated with lower incidence of poor QoL, while increased Physiologic Health score was associated with an elevated incidence of poor QoL (OR: 1.16; 95%CI: 1.06–1.27; *P* = 0.001). A prediction model for QoL based on these factors was constructed, and the AUC was 0.96 (95% CI: 0.94–0.98) (Fig. 1).

**Table 2:** Univariate and multivariate logistic regression for QoL

Variable	Univariate		Multivariate	
	OR and 95%CI	*P*	OR and 95%CI	*P*
**Age (years)**				
< 50.0				
50.0–65.0	1.45 (0.57 ~ 3.70)	0.436	0.02 (0.00 ~ 0.15)	< 0.001
> 65.0	2.07 (0.79 ~ 5.39)	0.137	5.88 (0.99 ~ 33.33)	0.052
**Sex**				
Male				
Female	4.62 (1.64 ~ 13.05)	0.004	10.91 (1.24 ~ 96.14)	0.031
**Educational status**				
Primary schools				
Junior high school	0.77 (0.46 ~ 1.28)	0.313	0.10 (0.03 ~ 0.33)	< 0.001
High school or above	0.61 (0.34 ~ 1.11)	0.108	0.24 (0.03 ~ 1.52)	0.128
**Residential location**				
Village				
Cities and towns	0.74 (0.46 ~ 1.18)	0.207	0.07 (0.02 ~ 0.37)	0.002
**Occupation**				
Farmer				
Worker	0.47 (0.27 ~ 0.82)	0.008		
Other	1.65 (0.67 ~ 4.08)	0.274		
**Marital status**				
Married				
Other	0.07 (0.00 ~ 0.35)	< 0.001		
**Smoking**				
No				
Yes	0.69 (0.25 ~ 1.90)	0.476		
**Alcohol**				
No				
Yes	0.92 (0.50 ~ 1.67)	0.776	4.55 (1.32 ~ 14.29)	0.017
**Surgery approach**				
Total laryngectomy				
Partial laryngectomy	0.61 (0.39 ~ 0.97)	0.038	0.10 (0.03 ~ 0.41)	0.001
The laryngoscope was removed	0.77 (0.29 ~ 2.04)	0.592	0.09 (0.01 ~ 1.02)	0.053
**The escrow**				
No				
Yes	0.97(0.38 ~ 2.48)	0.948		
Physiologic Health	1.02(1.00 ~ 1.04)	0.133	1.16(1.06 ~ 1.27)	0.001
Psychosocial Health	0.72(0.67 ~ 0.77)	< 0.001	0.48(0.39 ~ 0.58)	< 0.001
Health Knowledge & Behavior	0.87(0.83 ~ 0.90)	< 0.001	0.92(0.86 ~ 0.97)	0.006
Perceived Health	0.86(0.78 ~ 0.95)	0.003	0.36(0.25 ~ 0.52)	< 0.001

## Discussion

This study aimed to investigate the association between the postoperative core nursing outcomes and QoL in patients with LC after laryngectomy and to construct a prediction model for QoL. A total of 331 LC patients after laryngectomy were recruited, and the predictors for QoL included age 50.0–65.0 years, female sex, junior high school as the highest schooling level, residence in cities and towns, not consuming alcohol, partial laryngectomy, and the Physiologic Health, Psychosocial Health, Health Knowledge & Behavior, and Perceived Health scores. A prediction model constructed based on these predictors showed high predictive performance for poor QoL.

Several studies have investigated predictors of QoL in LC patients [13, 14]. Williamson et al. examined QoL in 41 patients and found that 4.9% reported poor QoL. Patients undergoing primary radiotherapy reported the best QoL, whereas the worst QoL was observed in patients undergoing chemoradiotherapy or combined surgical treatment and chemoradiotherapy [13]. Vilaseca et al. assessed QoL after transoral laser microsurgery in patients with LC and found that QoL could be affected by tumor location, adjuvant radiation, and neck dissection [14]. These studies mainly focus on the relationship between clinical characteristics of patients and their QoL. However, perioperative care such as airway management, psychological care, and swallowing training can also impact the postoperative recovery and QoL of patients. Moreover, the association of revised core nursing outcomes with QoL in patients with LC remains unclear. Hence, the current study aimed to identify the predictors of QoL in patients with LC including the core nursing outcomes.

The predictors we identified are similar to those identified in previous studies. We found a significant association between age, gender, and QoL, possibly due to variations in the severity of LC among patients of different age groups and genders. In addition, the significant impact of education level and patient’s residential environment on the QoL of LC patients can mainly be attributed to: (1) Patients with higher education levels may place more emphasis on health check-ups, resulting in milder conditions of LC when diagnosed; (2) Patients residing in urban areas have significantly better access to medical resources and a more favorable environment compared to rural areas, leading to higher levels of postoperative rehabilitation strategies and facilities, which greatly influence the patients’ QoL [12]. Consistent with previous study, this study also found alcohol consumption was associated with the QoL in LC patients undergoing total laryngectomy [15]. The surgical approach affects several domains of the revised core nursing outcomes. In previous studies, the physiological, overall functional, and head and neck functional statuses associated with partial laryngectomy were higher than those associated with total laryngectomy, especially in terms of appearance, vocalization, eating, and communication [16]. Moreover, partial laryngectomy preserves more sensory nerves in the skin and mucosa [17].

The revised postoperative core nursing outcomes reflects the use of nursing interventions in patients with LC, which is an important factor for improving their QoL [18]. Moreover, the revised postoperative core nursing outcomes mainly focuses on “Physiologic Health,” “Psychosocial Health,” “Health Knowledge & Behavior,” and “Perceived Health,” which mainly reflect the status of LC patients after laryngectomy and are significantly associated with QoL. Interesting, we noted higher Physiologic Health scores were associated with poorer QoL, which could explained by patients with higher Physiologic Health scores may have a heightened awareness of their physical limitations and symptoms, leading to a more negative perception of their overall QoL. These items in the core nursing outcome evaluation system for LC patients constructed were approved by clinical nurses, which was used to a certain extent in clinical nursing work of Otolaryngology, thus that the core nursing outcome with good clinical applicability. Therefore, targeted health education and behavior guidance based on revised core nursing outcomes to improve the QoL in LC patients, including: (1) providing individual and group counseling services to help patients cope with the emotional and psychological challenges associated with laryngectomy [19]; (2) establishing peer support programs where LC patients can connect with others who have undergone similar experiences [20]; (3) facilitating support groups that bring together LC patients and their families to share experiences, exchange information, and provide mutual support [21]; (4) incorporating mindfulness and relaxation techniques to help patients manage stress and improve their overall well-being [22]; and (5) Encouraging the involvement of family members in the care process, providing them with education and support to better understand and assist their loved ones [23].

LC surgery may entail physiological alterations such as changes in voice, difficulty in swallowing, and modifications to breathing patterns, as well as external and functional changes necessitated by the use of tracheostomy tubes or electronic speech devices. These alterations can significantly impact the patient’s mental well-being and prospects for reemployment [24, 25]. Therefore, constructing a predictive model for postoperative QoL in LC patients holds significant clinical importance. Our study constructed a prediction model based on identified predictors showed high predictive value for QoL. Thus, the revised core nursing outcomes provide a good foundation for the used in clinical practice, and multidimensional nursing interventions should be applied to further improve QoL.

This study has several limitations. First, as the sample includes patients from three hospitals, there is a possibility of selection bias. Patients from other regions or healthcare facilities may exhibit different predictors of QoL due to variations in care practices. Second, the cross-sectional design limits the ability to establish causality. Prospective or longitudinal studies would be better suited to confirm whether the identified factors genuinely impact QoL over time. Third, factors such as socioeconomic status, comorbidities, stage of LC, prior treatments (e.g., chemoradiotherapy), and access to rehabilitation services were not included in the analysis, yet these factors could significantly impact QoL of patients with LC. Fourth, the sample was predominantly male and smokers, reflecting the typical demographic for LC but potentially skewing the findings. Including a more gender-balanced sample might yield different insights, particularly given that gender was an influential factor. Fifth, LC patients were obtained in northern and northeastern China, which may limit the generalizability of our findings. Sixth, QoL was assessed using self-reported questionnaires, which are subject to response and recall biases. This can impact the accuracy of data on subjective measures like psychosocial health.

## Conclusions

In summary, age, sex, educational levels, residential status, alcohol consumption, surgical type, and Physiologic Health, Psychosocial Health, Health Knowledge & Behavior, and Perceived Health scores are significantly associated with the QoL in LC patients. Moreover, a prediction model based on these predictors had a high predictive value. Further prospective study should be performed to validate predictive model in diverse settings.

## Data Availability

The datasets used and/or analysed during the current study are available from the corresponding author on reasonable request.

## Abbreviations

LC: Laryngeal carcinoma
QoL: Quality of life
NOC: Nursing outcomes classification
FACT-H&N: Functional assessment of cancer therapy-head and neck
ORs: Odds ratios
CIs: Confidence intervals
AUC: Area under the receiver operating characteristic curve
